# Allogeneic bone marrow mesenchymal stem cell-derived exosomes alleviate human hypoxic AKI-on-a-Chip within a tight treatment window

**DOI:** 10.1186/s13287-024-03674-8

**Published:** 2024-04-10

**Authors:** Sefa Burak Çam, Eda Çiftci, Nazlıhan Gürbüz, Bülent Altun, Petek Korkusuz

**Affiliations:** 1 Faculty of Medicine, Dept. of Histology and Embryology, Hacettepe University, Ankara, Ankara 06230 Turkey; 2 Graduate School of Science and Engineering, Department of Bioengineering, Hacettepe University, Ankara, 06230 Turkey; 3 Faculty of Medicine, Dept. of Nephrology, Hacettepe University, Ankara, 06230 Turkey

**Keywords:** Ischemic AKI, Proximal tubule, Exosome, Kidney-on-a-chip, Hypoxia, Mesenchymal stem cells

## Abstract

**Background:**

Acute hypoxic proximal tubule (PT) injury and subsequent maladaptive repair present high mortality and increased risk of acute kidney injury (AKI) - chronic kidney disease (CKD) transition. Human bone marrow mesenchymal stem cell-derived exosomes (hBMMSC-Exos) as potential cell therapeutics can be translated into clinics if drawbacks on safety and efficacy are clarified. Here, we determined the real-time effective dose and treatment window of allogeneic hBMMSC-Exos, evaluated their performance on the structural and functional integrity of 3D microfluidic acute hypoxic PT injury platform.

**Methods:**

hBMMSC-Exos were isolated and characterized. Real-time impedance-based cell proliferation analysis (RTCA) determined the effective dose and treatment window for acute hypoxic PT injury. A 2-lane 3D gravity-driven microfluidic platform was set to mimic PT in vitro. ZO-1, acetylated α-tubulin immunolabelling, and permeability index assessed structural; cell proliferation by WST-1 measured functional integrity of PT.

**Results:**

hBMMSC-Exos induced PT proliferation with ED50 of 172,582 µg/ml at the 26th hour. Hypoxia significantly decreased ZO-1, increased permeability index, and decreased cell proliferation rate on 24–48 h in the microfluidic platform. hBMMSC-Exos reinforced polarity by a 1.72-fold increase in ZO-1, restored permeability by 20/45-fold against 20/155 kDa dextran and increased epithelial proliferation 3-fold compared to control.

**Conclusions:**

The real-time potency assay and 3D gravity-driven microfluidic acute hypoxic PT injury platform precisely demonstrated the therapeutic performance window of allogeneic hBMMSC-Exos on ischemic AKI based on structural and functional cellular data. The novel standardized, non-invasive two-step system validates the cell-based personalized theragnostic tool in a real-time physiological microenvironment prior to safe and efficient clinical usage in nephrology.

**Supplementary Information:**

The online version contains supplementary material available at 10.1186/s13287-024-03674-8.

## Background

Acute hypoxic proximal tubule (PT) injury is the initiation step of ischemic acute kidney injury (AKI) [1] presenting a high risk for chronic kidney disease (CKD) [2, 3] on hospitalized and intensive care unit patients [4, 5]. As a preventable cause of morbidity and mortality, extensive research is needed for prevention of AKI as underlined by International Society of Nephrology (ISN)’s 0by25 initiative [6]. Current experimental models for research involving cell monolayers [7–14] and in vivo animal settings [15–18] evaluate hypoxic PT injury for theragnostic approaches to some extent. The human monolayer renal parenchymal cell cultures even though easy to set [19], provide poor epithelial polarization that may cause low levels of key protein transporters [20], no output on PT ultrafiltrate flow [21] and do not contain any of the stromal microenvironmental elements [22]. Murine models, mainly consisting of surgical ischemia protocols, are able to monitor kidney functions within in vivo organ/system configuration to investigate efficacy and safety of therapeutic candidates [19, 23–26]. However; murine PT hypoxic injury differs from humans in terms of cellular responses [27] relating to different pharmacokinetic profiling of chemical and cellular therapeutics [28, 29] alongside ethical concerns [30] for clinical scaling. The cell-based agents require safety assessment and efficacy standardization before clinical use as therapeutic candidates [31, 32]. 

Mesenchymal stem cells (MSC) [33, 34] and MSC exosomes (MSC-Exos) [27, 35] are strong therapeutic candidates for AKI but their safety [36, 37] and efficiency [38, 39] remain controversial. A total of 8 currently undergoing or completed phase I-II clinical trials with MSC-based therapeutics targeting post-operative or drug-induced AKI [40] reported limited outputs with only one completed study [41] and no study on MSC-Exos exists. Allogeneic and xenogeneic MSC-Exos gave favorable outcomes in terms of increasing epithelial cell proliferation [42], reducing cell death [42–44] and injury biomarkers [45] when applied to monolayer acute hypoxic PT injury setups [44–49]. They induce homing to kidney [42, 50], improve serum creatinine [23, 42, 43], blood urea nitrogen [23, 43] levels and reduce cell death [23, 43, 51] when applied to murine acute surgical ischemia models [24, 42, 49, 51–54] for AKI. Regarding these favorable results, MSC-Exos may be key to preventing maladaptive repair of PT epithelium in ischemic AKI to deter AKI - CKD transition [3, 55, 56]. The lagged transition of these expectations to clinic may be due to the uncertainties of safe dose range (10 to 1000 µg/ml) [23, 45, 57], treatment window (0 to 48 h) [46, 58] and heterogenous population (30 to 200 nm) of extracellular vesicles [36, 39]. Exosomes require preclinical real-time standardized potency assessment following proper expansion, characterization and purification for translation as potential precision medicine tools.

Three-dimensional (3D) microfluidic proximal tubule-on-a-chip platforms are fine-tunable standardized dynamic ex vivo microenvironments that provide tubular epithelial cell polarization, fluid shear stress, reabsorption, secretion capability and peritubular vascularization in physiological and pathological conditions [59–64]. Membraneless microphysiological systems with peristaltic pumps and tubing sensitively screened multiple (1–4 to 9) nephrotoxic agents in one setting in terms of heme oxygenase-1 (HO-1) and kidney injury molecule-1 (KIM-1) as potential biomarkers for AKI [65–67]. Polydimethylsiloxane (PDMS)-based pumped systems assess cell proliferation, apoptosis and PT epithelial functions such as albumin uptake, ALP activity and glucose transport [68] for drug-induced AKI. Those prototypes require pumps and external tubing for each chip, thus lacking ease of use in routine assays of PT epithelium. In recent studies, gravity-driven platforms without an artificial membrane screened 3D PT structure by zonula occludens 1 (ZO-1) and acetylated α-tubulin immunostaining [69–71], cellular proliferation assays [69, 71–74] and epithelial barrier integrity assays [69, 73, 74] for drug-induced AKI. These microphysiological systems may real-time evaluate cellular therapeutics for acute hypoxic PT injury in AKI [61, 75–77]. 

In this study, we hypothesized that a gravity-driven membraneless microfluidic-based 3D culture platform can reproduce acute hypoxic PT injury and real-time assess the therapeutic potency of human bone marrow-derived MSC exosomes (hBMMSC-Exos) as cellular therapeutics. To test this hypothesis, we precisely isolated, purified, characterized, and quantitatively analyzed hBMMSC-Exos with a novel proliferative potency assay in acute hypoxic PT injury in vitro. A gravity-driven 2-lane microfluidic PT-on-a-chip platform modeled the hypoxic PT injury ex vivo in terms of structure and function by epithelial permeability, cell polarity, and proliferation. The novel microfluidic system successfully established acute hypoxic PT injury and assessed the therapeutic potential of allogeneic hBMMSC-Exos in terms of structural integrity and functional capacity.

## Methods

### Study design

An observational, prospective in vitro study was designed with the independent variables of the groups (a) 2D-Normoxia, (b) 2D-Hypoxia, (c) 3D-Normoxia, (d) 3D-Hypoxia, (e) 3D-Hypoxia + Vehicle and (f) 3D-Hypoxia + hBMMSC-Exos, respectively.

### Construction of Acute hypoxic PT Injury-on-a-Chip, Ex Vivo Assessment of Acute Hypoxic PT Injury and therapeutic potential of hBMMSC-Exos

#### Cell culture

HK-2, human immortalized PT epithelial cell line (CRL-2190, ATCC-LGC, USA) was cultured in Keratinocyte-Serum Free Medium (17,005,042, Thermo, USA) with 5 ng/ml of human recombinant epidermal growth factor, 0.05 mg/ml of bovine pituitary extract. The cells were tested for mycoplasma contamination, examined in transmission electron microscopy (TEM) to validate their cellular characteristics and used in passages 8–10.

#### Microfluidic chip setup

A commercial organ-on-a-chip system composed of microfluidic culture plates containing 96 individual membraneless chips with 2 microchannels were utilized to model 3D PT epithelium in microphysiological conditions (2-lane OrganoPlate, Mimetas, Netherlands). Briefly, 2 µl of extracellular matrix (ECM, Matrigel, Corning, USA) was pipetted to gel microchannel and polymerized at 37°C for 30 min for each chip, and 1.5 × 10^4^ cells in 2 µl of media were seeded to flow microchannel. An intermittent rocker platform (OrganoFlow L, Mimetas, Netherlands) provided perfusion of the chips with a swing angle of (-7°) – (+ 7°) and an interval of 8 min.For 2D control, 1.5 × 10^4^ cells in 200 µl media were seeded to 96-well flat-bottom plates.

#### Hypoxia setup

A standard protocol based on a gas pressure change in the incubator environment (Panasonic, Japan) was applied to mimic hypoxic injury [78]. The samples were cultured at 37°C with either hypoxia (1% O_2_, 5% CO_2_) or normoxia (17% O_2_, 5% CO_2_). O_2_ concentration was monitored with a zirconium O_2_ sensor of the incubator (Panasonic, Japan).

#### Cellular proliferation assay

The effects of the acute hypoxic PT injury on the proliferation of PT cells were assessed with WST-1 assay (ab155902, Abcam, UK) with a colorimetric plate reader.

#### Epithelial barrier integrity assay

Fluorescein isothiocyanate (FITC)-dextran and tetramethyl rhodamine (TRITC)-dextran probes weighing 20 kDa and 155 kDa were dissolved in culture media with 0.5 mg/ml concentration each. After perfusion leakage of the probes into the ECM was quantified in series of fluorescent micrographs under fluorescent microscope (IX-73, Olympus, Japan). The permeability index of the epithelial barrier was calculated for each chip from the intensity data acquired according to the formula.

P_app_ (cm/s) = (ΔC_receiver_ x V_receiver_) / (Δt x A_barrier_ x C_donor_)

#### Immunolabelling

As previously described [69, 79], the cells were fixed within 3.7% paraformaldehyde, permeabilized with 0.1% Triton X-100, blocked with 2% BSA and incubated with Alexa Fluor 594 conjugated ZO-1 (339,194, Invitrogen, USA) and FITC conjugated acetylated α-tubulin (sc-23,950, Santa Cruz, ABD) antibodies at 4°C overnight. DAPI stained the nuclei (D1306, Thermo, USA). Micrographs were captured with fluorescent (IX-73, Olympus, Japan) or confocal microscope (DM8i, Leica, Germany) to determine means of corrected total fluorescence intensity (CTFI) of the immunolabelling with ImageJ software according to the formula below.

CTFI = [Integrated Density – (Area of ROI x Mean Background)] / Total Cell Count

### Isolation, characterization, and proliferative potency assay of hBMMSC-Exos

The exosome isolation and characterization have been performed in total accordance to “Minimal information for studies of extracellular vesicles 2018 (MISEV 2018)” guidelines [80] with sequential ultracentrifugation followed by protein concentration, morphology (size and its distribution) and at least 2 out of 3 CD markers (CD63 and CD81).

#### Cell culture

hBMMSCs (PCS-500-012, ATCC, USA) were cultivated in Dulbecco’s Modified Eagle Medium (DMEM) with 10% fetal bovine serum (FBS) and 1% pen-strep at 5% CO_2_ and 37 °C. The cells were tested for mycoplasma contamination and experiments were conducted in passage 4.

#### Ultracentrifugation

hBMMSCs were washed with phosphate-buffered saline (PBS) and cultivated at 5% CO_2_ and 37 °C in DMEM supplemented with exosome-depleted FBS (10%) for 48 h. The collected media was centrifuged at 1500xg for 10 min, 10000xg for 10 min, and 30000xg for 30 min, respectively. The supernatant was filtered with 0.22 micron filter and ultracentrifuged for 2 × 90 min at 100000xg. The collected exosomes were resuspended in PBS and stored at -80 °C.

#### TEM and BCA

Bicinchoninic acid (BCA) protein quantification and TEM were performed to characterize hBMMSC-Exos [81]. BCA assay (#23,225, Thermo, USA) was performed and measured with a colorimetric microplate reader in 562 nm (VersaMax, Molecular Devices, USA).

For TEM, hBMMSC-Exos were precipitated onto 200 mesh formvar/carbon-coated nickel grids (EMS, USA) by gravity and stained with 1% phosphotungstic acid and 2% uranyl acetate. Grids were air-dried and inspected by TEM (JEM 1400, JEOL, Japan). hBMMSC-Exos were quantitatively evaluated for width and length in micrographs captured with a digital camera (Gatan, USA).

#### Flow cytometry

The hBMMSC-Exos were characterized by their surface markers CD63 and CD81 using flow cytometry. First, exosomes were captured with CD63-conjugated capture beads (ab239686, abcam, USA) and labeled with phycoerythrin (PE)-conjugated CD81 antibody (130-118-481, Miltenyi, USA) after serum blockage. CD63 and CD81 positive exosomes were quantified using a flow cytometer (Novocyte 2000, Agilent, USA) against isotype control.

#### RTCA

Real-time impedance-based cell proliferation analysis (RTCA, Agilent, USA) quantitatively determined the effective therapeutic dose (ED50) and time profile of hBMMSC-Exo intervention. HK-2 cells were plated in 96-well E-plates as 1.5 × 10^4^ cells/well [79]. hBMMSC-Exos were applied under normoxia (17% O_2_) with doses of 0.5 µg/ml, 5 µg/ml, 50 µg/ml, and 500 µg/ml, respectively to determine the treatment window. A time-dependent ED50 graph determined the time profile of hBMMSC-Exos intervention. The ED50 of hBMMSC-Exos in acute hypoxic PT injury was determined with application of the same hBMMSC-Exos doses under hypoxia (1% O_2_) for 48 h.

### Statistics

The Shapiro-Wilk test assessed the normality of distribution. The subsequent parametric data were evaluated with one-way variance analysis and post hoc Tukey test. Kruskal-Wallis and post hoc Mann Whitney U tests were conducted for the analyses of the nonparametric data. All analyses were plotted with GraphPad Prism8 (v8.4.2, USA) with a significance degree of *p* < 0.05.

## Results

### Acute hypoxic PT Injury impairs structural and functional integrity of the epithelium on 3D dynamic microfluidic setup

**Fig. 1:**
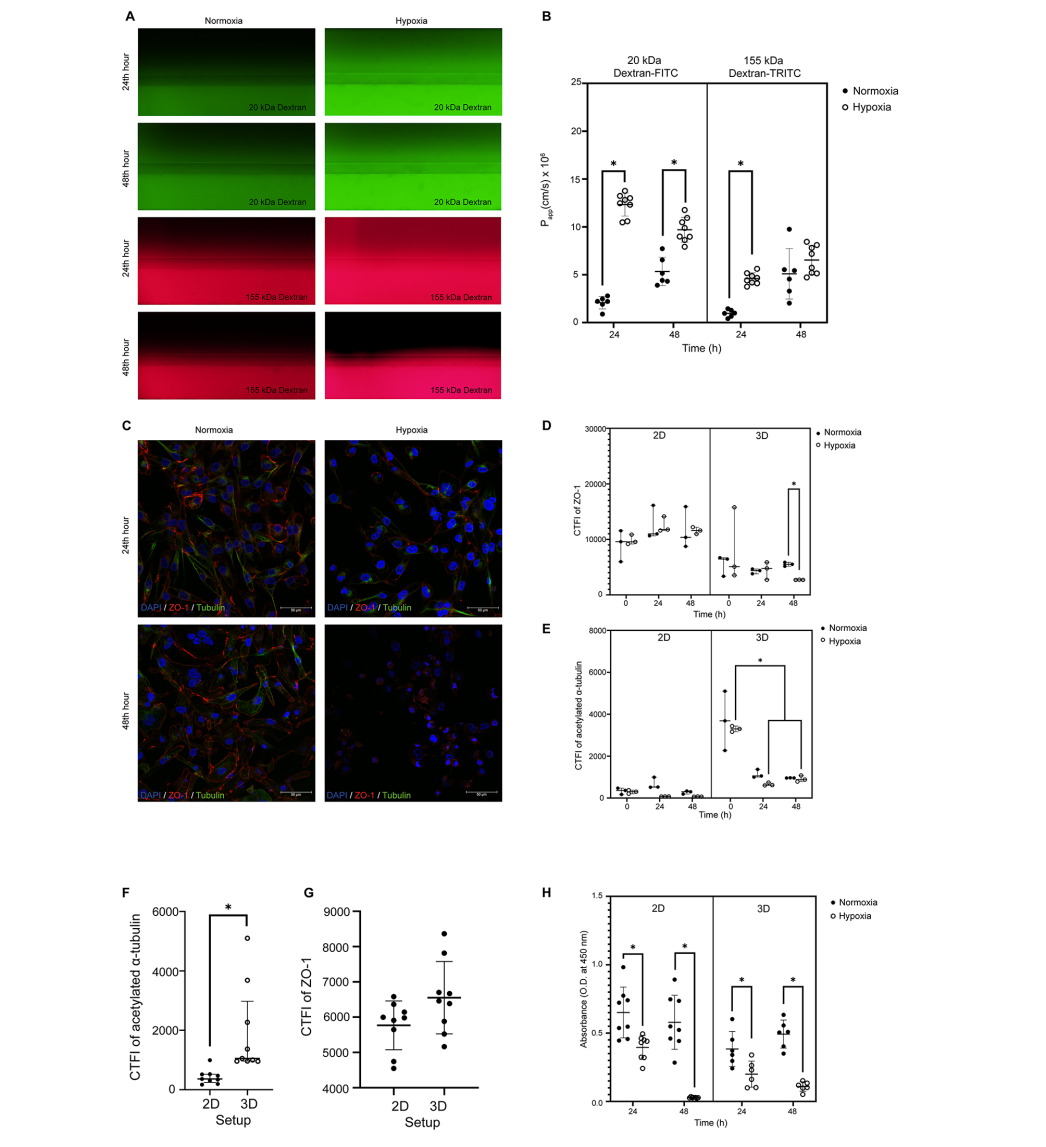
The epithelial barrier integrity, polarity, and PT cell proliferation deteriorate upon acute hypoxic tubular injury. **(A)** The barrier integrity assay revealed increased permeability for the 3D-Hypoxia group against both 20 kDa- and 155 kDa-Dextran. (*n* = 28) **(B)** Permeabilization indexes revealed increased permeability for the 3D-Hypoxia group against 3D-Normoxia for both 20 kDa- and 155 kDa-Dextran. (*n* = 28) **(C)** ZO-1 (Alexa Fluor 594) and acetylated α-tubulin (FITC) immunolabelling showed structurally intact and polarized PT epithelial cells in 2D- and 3D-Normoxia groups compared to the loss of polarity in 2D- and 3D-Hypoxia groups. (*n* = 36) **(D)** The CTFI detected a decrease in ZO-1 immunolabelling compared to 3D-Normoxia in 48 h compared to 0–24 h. (*n* = 36) **(E)** Calculated CTFI revealed a decline in acetylated α-tubulin immunolabelling in 3D-Hypoxia in 48 h. **(F)** The CTFI detected an increase in acetylated α-tubulin immunolabelling in 3D-Normoxia compared to the 2D-Normoxia, indicating amplification of polarization with fluid flow. (*n* = 18) **(G)** The CTFI of ZO-1 was similar in 2D and 3D setups in normoxia. (*n* = 18) **(H)** WST-1 assay detected a decrease in proliferation rate of PT cells in 2D-Hypoxia compared to 2D-Normoxia in both 24 and 48 h. The proliferation rate of 2D-Hypoxia was similar to 3D-Normoxia in 24 h and was decreased at 48 h. (*n* = 56) Data in scattered dot plots **(B)**, **(D-E)** and **(G-H)** are mean ± SD. Data in scattered dot plot **(F)** are median ± interquartile range. (*) denotes (*p* < 0.05) comparing the groups in the indicated time point

Proximal tubule epithelial cells formed 3D tubules successfully on day 8 in the microfluidic system under normoxia validated by a phase-contrast microscope. The barrier integrity assay revealed increased PT permeability by 5.92- and 4.80-fold against 20 kDa and 155 kDa dextran respectively under hypoxia compared to the normoxia at 24 h (*p* = 0.0001 and *p* = 0.0001, respectively; Fig. 1A and B). Permeability against 20 kDa dextran remained 1.81-fold high under hypoxia compared to normoxia at 48 h (*p* = 0.0001, Fig. 1A and B). Permeability against 155 kDa dextran was slightly recovered under hypoxia giving a similar P_app_ compared to normoxia at 48 h (*p* > 0.05, Fig. 1B).

Acute hypoxic PT injury decreased the CTFI of ZO-1 immunolabelling by 50% at 48 h in 3D setups compared to normoxia (*p* = 0.0131, Fig. 1C and D). The 3D setups showed similar ZO-1 expressions in both normoxia and hypoxia at 0 and 24th hours (*p* > 0.05, Fig. 1C and D). PT epithelial cells in 2D and 3D setups presented granular labeling patterns for ZO-1 under hypoxia, unlike the continuous reticular appearance of intercellular tight junctions under normoxia (Fig. 1C). Comparison of CTFI revealed similar intensity levels of ZO-1 in 2D setups under hypoxia and normoxia from 0 to 48 h (*p* > 0.05 for each time point, Fig. 1D).

Acetylated α-tubulin expression of PT epithelial cells was diminished by 80% and 72% in time from 0 to 24 and 48 h respectively under hypoxia compared to initial expression in the 3D setup in terms of CTFI (*p* = 0.003 and *p* = 0.0023, respectively, Fig. 1C and E). Acetylated α-tubulin showed similar expressions in 2D samples under both normoxia and hypoxia throughout all time points in terms of CTFI (*p* > 0.05 for each time point, Fig. 1C and E). The 3D microfluidic setup enhanced the expression of acetylated α-tubulin in terms of CTFI under normoxia in all time points compared to the 2D setup (*p* = 0.0003, Fig. 1F). The expression of ZO-1 was similar in both 2D and 3D setups in terms of CTFI (*p* > 0.05, Fig. 1G).

Acute hypoxia decreased the proliferation rate of PT cells by 39% and 95% respectively compared to normoxia in 2D setups at both 24 (*p* = 0.0026) and 48 h (*p* = 0.0001, Fig. 1H). The proliferation rate also declined 48% and 77% respectively in 3D setups under hypoxia compared to normoxia at 24 (*p* = 0.0069) and 48 h (*p* = 0.0001, Fig. 1H).

### hBMMSC-Exos induce PT cellular proliferation under both normoxia and hypoxia with an ED50 of 172,582 µg/ml at 26th hour

**Fig. 2:**
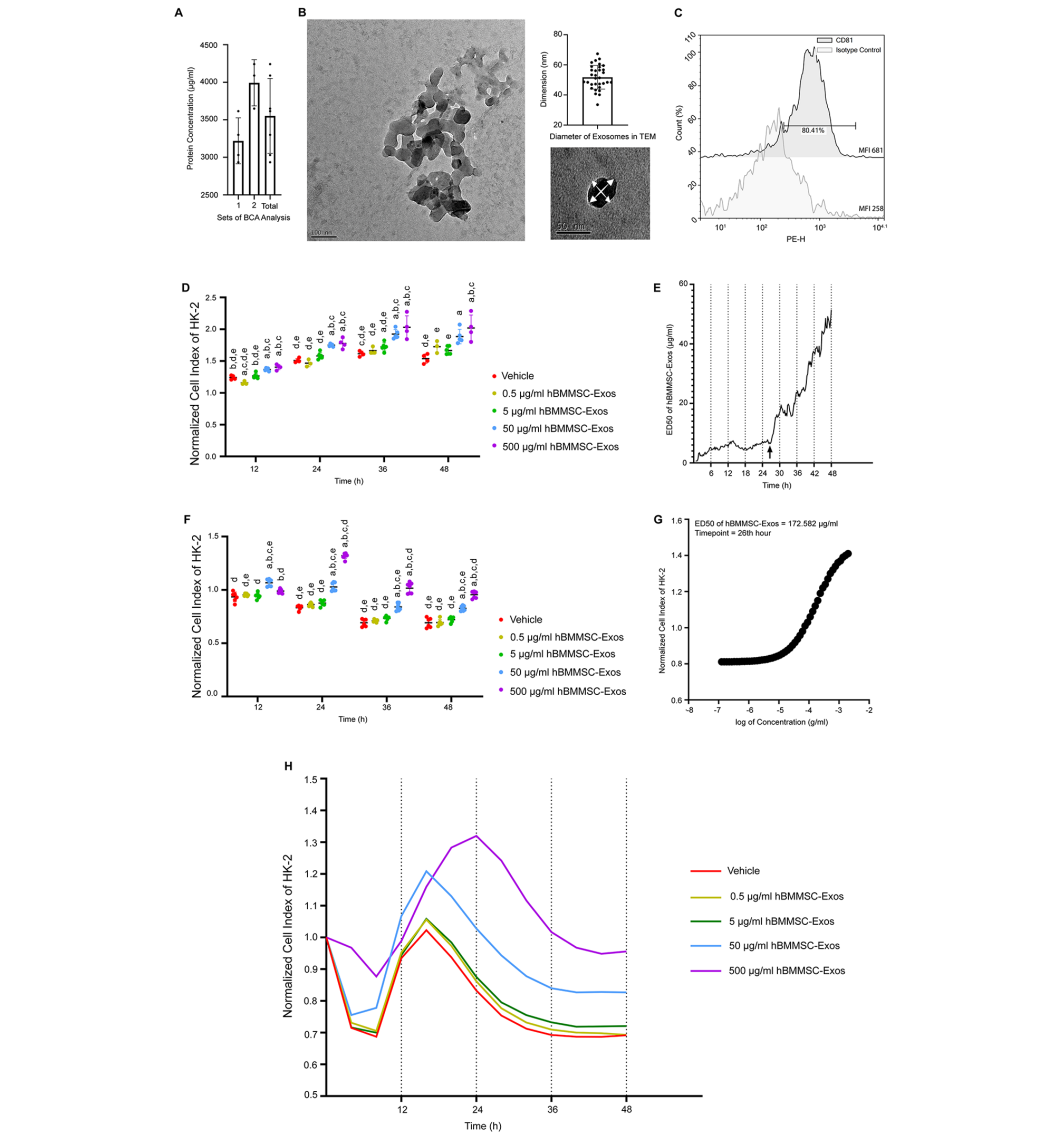
Isolated and characterized hBMMSC-Exos induce PT cellular proliferation under normoxia and hypoxia. **(A)** BCA assay detected a high protein concentration of hBMMSC-Exos samples (*n* = 7). **(B)** Electron micrographs of aggregated and individual hBMMSC-Exos revealed homogenous and typical globular particles. Micrographs were also quantified in terms of diameter and size distribution was demonstrated as scatter plot. Uranyl acetate, phosphotungstic acid; 150000x (Scale bar = 100 nm), 250000x (Scale bar = 50 nm) (*n* = 32). **(C)** Flow cytometry revealed that 80.41% of exosomes were positive for surface marker CD81 compared to isotype control. (*n* = 2) **(D)** The normalized cell index scatter dot plots from 12 to 48 h and **(E)** ED50 to time graphic of RTCA data for hBMMSC-Exos treatment on PT epithelial cells under normoxia (*n* = 22). Arrow marks 26th hour of the experiment as the end of the treatment window. **(F)** The normalized cell index scatter dot plots from 12 to 48 h, **(G)** dose-response curve and **(H)** normalized cell index to time graphic of RTCA data for hBMMSC-Exos treatment on PT epithelial cells under hypoxia (*n* = 32). ED50 of hBMMSC-Exos on PT epithelial cells at 26th hour was calculated as 172,582 µg/ml. The descriptive data is plotted as mean ± SD in **(A)**, **(D)** and **(F)**. (a) denotes p˂0.05 compared to vehicle, (b) to 0.5 µg/ml hBMMSC-Exos, (c) to 5 µg/ml hBMMSC-Exos, (d) to 50 µg/ml hBMMSC-Exos and (e) to 500 µg/ml hBMMSC-Exos groups

The BCA assay detected the mean protein concentration as 3552 ± 499.1 µg/ml for hBBMSC-exos (Fig. 2A). Aggregated or individual exosomes presented as typical spherical (*p* > 0.05) nano-sized intact particles in a homogenous size range between 33.55 and 67.33 nm under TEM (Fig. 2B). Flow cytometry revealed that 80.41% of exosomes were positive for surface marker CD81 and mean fluorescent intensity of the labeling was 2.63-fold higher compared to isotype control (*p* < 0.0001, Fig. 2C).

Proliferative potency assay revealed that hBMMSC-Exos increased the proliferation rate of PT cells compared to vehicle at 50 µg/ml and 500 µg/ml doses from 12 to 48 h under normoxic conditions (*p* = 0.0003 and *p* = 0.0001 for 12 h, *p* = 0.0001 and *p* = 0.0001 for 24 h, *p* = 0.021 and *p* = 0.0132 for 36 h, *p* = 0.0028 and *p* = 0.0002 for 48 h; Fig. 2D). The 26th hour of the experiment, where ED50 began to elevate significantly, marked the decline in the proliferative potency of the particles and indicated the end of treatment window (Fig. 2E). The effective proliferative dose of hBMMSC-Exos on HK-2 cells was determined as 7.0409 µg/ml at the 26th hour under normoxia (R^2^ = 0.99926).

hBMMSC-Exos increased the proliferation rate of PT cells compared to vehicle at 50 µg/ml and 500 µg/ml doses from 12 to 48 h under hypoxia (*p* = 0.0001 and *p* = 0.0215 for 12 h, *p* = 0.01 and *p* = 0.0001 for 24 h, *p* = 0.0001 and *p* = 0.0001 for 36 h, *p* = 0.0001 and *p* = 0.0001 for 48 h; Fig. 2F and H). The effective proliferative dose of hBMMSC-Exos on HK-2 cells was determined as 172.582 µg/ml at the 26th hour under hypoxia (R^2^ = 0.99999, Fig. 2G).

### hBMMSC-Exos alleviate hypoxic injury on barrier integrity and cellular proliferation

**Fig. 3:**
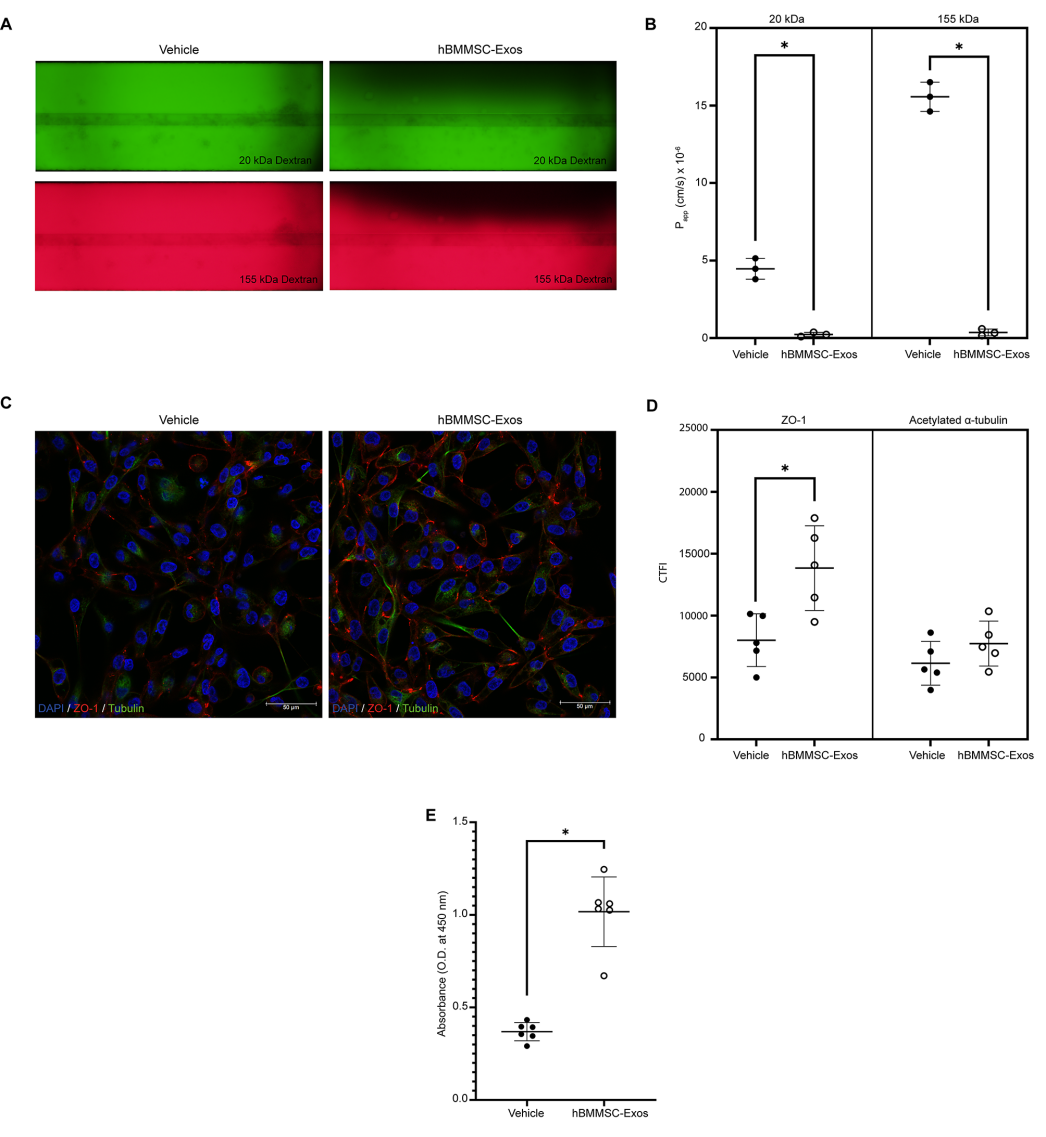
hBMMSC-Exos alleviate hypoxic injury of PT cells by decreasing epithelial permeability, increasing proliferation and polarity. **(A)** hBMMSC-Exos decreased the permeabilization of epithelial barrier against 20 kDa and 155 kDa Dextran. (*n* = 6) **(B)** The barrier integrity assay revealed amelioration of hypoxic injury on PT epithelial barrier upon 24th hour after hBMMSC-Exos treatment. (*n* = 6) **(C)** ZO-1 (Alexa Fluor 594) and acetylated α-tubulin (FITC) immunolabelling showed structurally intact and polarized PT epithelial cells in hBMMSC-Exos group compared to the loss of polarity in vehicle. (*n* = 10) **(D)** CTFI outputs revealed the increase of ZO-1 after hBMMSC-Exos treatment reversing the effects of hypoxic injury. (*n* = 10) **(E)** hBMMSC-Exos increased the proliferation rate of PT epithelial cells after acute hypoxic tubular injury (*n* = 12). Plotted data in scatter dot plots **(C)**, **(D)** and **(E)** are mean ± SD. (*) denotes (*p* < 0.05) between indicated groups

hBMMSC-Exos alleviated hypoxic injury in terms of barrier integrity 20-fold against 20 kDa dextran (*p* = 0.0004) and 45-fold against 155 kDa dextran (*p* = 0.0001) compared to the 3D-Hypoxia-Vehicle group in 24 h after treatment (Fig. 3A and B). CTFI of ZO-1 expression was significantly increased after hBMMSC-Exo treatment in injured PT epithelial cells (*p* = 0.0121, Fig. 3C and D). hBMMSC-Exos did not alter the CTFI of acetylated α-tubulin (*p* > 0.05, Fig. 3C and D). hBMMSC-Exos treatment improved the proliferation rate of PT epithelial cells compared to the vehicle group (*p* = 0.0001, Fig. 3E).

## Discussion

In this report, we generated a new human 3D gravity-driven microfluidic acute hypoxic tubular injury platform that assessed structural and functional phenotype of PT epithelial cells under hypoxic conditions, and subsequently evaluated the therapeutic performance of allogeneic hBMMSC-Exos on the validated ischemic AKI model. A novel real-time potency assay established precise proliferative ED50 and treatment window of hBMMSC-Exos as 172.582 µg/ml for 26 h for ischemic AKI. The microfluidic platform successfully validated the treatment window for allogeneic hBMMSC-Exos correcting PT epithelial barrier and restoring cellular polarity by ZO-1 for 24 h.

We first constructed the non-invasive *ex-vivo* 3D gravity-driven microfluidic acute hypoxic tubular injury platform to mimic injury process on human PT in AKI. Here, we achieved the formation of the PT epithelial barrier by leak-tight tubules from day 8 under normoxia. Previous studies report the establishment of the barrier in a period between 7 and 10 days for drug-induced AKI models within the same microfluidic platform [69, 71]. PT epithelial barrier that is successfully formed on day 8 in our study confirmed the standard performance of the system. Then, we established acute hypoxic PT injury by setting the 3D microfluidic platform into a 1% oxygenated incubator for 48 h. Acute hypoxia increased PT epithelial permeability, decreased polarity and decreased epithelial proliferation rate from 24 to 48 h within the 3D microfluidic system. The hypoxia and its structural/functional impacts on ischemic AKI have not been assessed in any 3D platform before.

Acute hypoxia decreased PT epithelial cell numbers by 49% at 24 h and 78% at 48 h in the 3D microfluidic platform; similar to 40% at 24 h and 97% at 48 h in 2D culture. The previous acute hypoxia models set by 1% oxygenation on 2D monolayer culture revealed a decrease in proliferation rate in a range of 30–95% [9, 12–14] at 24 h and 65–99% [14, 82] at 48 h by MTT/MTS assays. 3D microfluidic systems similarly detected a decrease in proliferation rate of PT cells in a range of 10–99% in drug-induced AKI models by WST-8 [69, 72–74]. Our data related to proliferation decrease on 2D monolayer controls were totally in line with previous literature and our 3D microfluidic platform have been successful to sense the decrease in epithelial cells and screen acute hypoxic injury. However, proliferation rate stands for late phases of cell loss which is anticipated by impairment of epithelial junctions causing functional incapability. 3D microfluidic proximal tubule-on-a-chip platforms might monitor those early phases of epithelial impairment.

Here we describe that 3D microfluidic platform-based hypoxia displayed a significant increase in PT epithelial permeability by 6- and 4-fold against 20 kDa and 155 kDa dextran at 24 h, respectively, and by 2-fold against 20 kDa dextran at 48 h compared to normoxia. The selected dextran probe molecular weights of 20 and 155 kDa represent small molecules passing through glomerular filtration barrier (GFB) in physiological conditions and large molecules leaking through GFB reflecting pathological conditions such as AKI, respectively [83]. Epithelial permeability has been studied on 3D microfluidic systems before in drug-induced AKI models [69, 73, 84, 85] but a study on ischemic AKI was missing. Our microfluidic setup defined the increase in epithelial permeability quantitatively in acute hypoxic PT injury in 3D microenvironment for the first time. We also report a 50% decrease in ZO-1 immunolabelling intensity and transition from a continuous reticular appearance to a granular labeling pattern reflecting the impairment of cellular junctions upon acute hypoxic PT injury at 48 h in 3D microfluidic platform. The 2D setup also revealed a granular pattern under confocal microscope but has not been sensitive enough to monitor the decline of ZO-1 labeling. Decrease in ZO-1 labeling is consistent with increased epithelial leakage by barrier integrity assay, which collectively refers to the impairment of tight junctions representing the key element of epithelial permeability [86]. Previous 2D [87] and 3D [74] in vitro studies reported decrease of ZO-1 intensity in a range of 20–90% upon or septic [87] or drug-induced [74] AKI. Our data of a 50% decrease in ZO-1 following hypoxic AKI at 48 h is coherent with the magnitude of decrease in earlier reports [74, 87] and marks the loss of epithelial polarity and integrity of tight junctions, leading to increased permeability. Here we report acute hypoxic PT injury decreased acetylated α-tubulin immunolabelling intensity by 80% and 70% at 24 and 48 h in 3D setup compared to 0 h. We also detected that the 3D microfluidic platform significantly increased acetylated α-tubulin intensity but not ZO-1 significantly compared to 2D setups under normoxia. The nonsignificant increase in ZO-1 is attributed to our limited sample size. Keele et al. [8] qualitatively reported that acetylated α-tubulin labeling in PT cells was increased upon shear stress via shaking on 2D setup and decreased under hypoxia (1% O_2_). Their monolayer setup on rocker presents similarity with our 3D microfluidic platform since they both include shear stress via perfusion and qualitative findings on acetylated α-tubulin labeling are parallel. In conclusion, our 3D microfluidic platform provided an optimal PT epithelial polarization with fluid flow and successfully assessed structural impairment due to acute hypoxia.

We isolated, and characterized hBMMSC-Exos for their morphology, protein quantification, and surface markers. Ultracentrifugation of hBMMSC media yielded a large amount (3694 ± 439.2 µg/ml) of hBMMSC-Exos as typical homogenous spherical vesicles with bilayer membrane under TEM with a size range of 33.55–67.33 nm in our study. Our exosome purification data confirmed optimal ultracentrifugation protocol which is compatible with our former work [88]. The previous studies on MSC-Exo treatments in ischemic AKI [17, 18, 42, 44, 48, 51] report a wide and heterogenous size ranging from 20 to 150 nm compared to our hBMMSC-Exos. The heterogeneity of MSC-Exo size in these studies can be attributed to different isolation protocols containing sequential ultracentrifugation + filtration [18, 42, 51], sequential ultracentrifugation alone [17, 44] and density gradient ultracentrifugation [48]. Our protocol of sequential ultracentrifugation + 0.22 μm filtration aimed to avoid cell debris and other extracellular vesicle types, resulting in a pure and homogenous vesicle population detected by TEM. We also characterized hBMMSC-Exos by high surface marker CD81 on flow cytometry as 80,41% compared to isotype control. The expression levels of CD81 in exosomes were previously reported in a range of 63-98.4% by flow cytometry [89–91] confirmed by high expression of CD81 proteins by western blot in several human MSC studies [18, 23, 92, 93]. Our data on the expression level of CD81 is coherent with the previous studies and validates isolation of the pure hBMMSC-Exos population. Thus, we confirmed the isolation of hBMMSC-Exos by their morphology, protein concentration, and surface marker CD81 as recently adopted triple characterization approach [94] in line with MISEV 2018 Guidelines [80] and our previous technical report outputs [88] assuring precise quantification of exosomes as potent therapeutic tools.

Prior to this study, exosomes lacked an established proliferative potency assay and had heterogeneous application reports in terms of dose and treatment window. The proliferative potency assay of hBMMSC-Exos revealed an effective proliferative dose of 172.582 µg/ml in ischemic AKI and a decrease in proliferative effect after 26th hour of the treatment. Previous potency assays of exosomes [95, 96] covered immunomodulatory effects of these nanovesicles but not cellular proliferation [97]. Earlier ischemic AKI studies used hBMMSC-Exos in a wide dose range of 10-1000 µg/ml with limited dose determination work [23, 45, 57]. We detected precise proliferative ED50 of hBMMSC-Exos within described dose range but at a short time window. Therefore our RTCA-based ED50 assessment sensing the temporal decline in proliferative efficacy of therapeutic agent manifests a novel strategy to estimate half-life of exosomes with a potential to overtake previous methods consisting of exogenous labeling [98], reporter proteins [99] and bioluminescence [100]. A recent study reported no difference in proliferation rates at 48 h and 2 weeks after 100 µg/ml of intravenous exosome application on an in vivo rat ischemic AKI setup [43]. Our previous study revealed the quick engraftment of fluorescent-labeled xenogeneic BMMSCs into rat proximal tubules (detected in 24 h, eliminated in 48 h) following renal arterial injection in ischemic AKI. Both studies revealing lack of effect and elimination of stem cells/stem cell exosomes within 48 h may appeal to the tight therapeutic window in our study. Therefore, real-time proliferative potency assay detecting ED50 in a tight time window presents a stepping stone for further in vivo preclinical treatment assessments and establishes the pharmacokinetics of exosomes as standardized therapeutics.

In our study, hBMMSC-Exos restored polarity and structural integrity by a 1.72-fold increase in ZO-1, and by a 20- and 45-fold decrease in permeability against 20 kDa and 155 kDa dextran, respectively. Although effects of hBMMSC-Exos on PT epithelial permeability is not studied before, exosomes derived from mouse BMMSCs [101] and human breast milk [102] increased ZO-1 immunolabelling in mouse brain microvascular endothelium and human intestinal epithelium, respectively. Those data are parallel with ours in terms of time window and beneficial effect of exosomes on integrity of epithelium but no dosing [101, 102]. Although previous nephrotoxicity studies in same 3D microfluidic platform successfully showed antiproliferative effects of applied therapeutics [66, 72, 103, 104], proliferative effect of hBMMSC-Exos was shown in a 3D platform for the first time. Therefore, we validated the determined dose and treatment window of hBMMSC-Exos in our 3D microfluidic setup, providing a novel assessment platform for therapeutics. Our results imply that hBMMSC-Exos could deter AKI-CKD transition by preventing maladaptive repair of the PT epithelium, increasing proliferation, and preserving cell polarity.

Our data are limited to in vitro and ex vivo conditions, requiring further confirmation in in vivo microenvironment to get a comprehensive grasp on ischemic AKI and safe use of hBMMSC-Exos in the nephrology clinic. Allogeneic cell-based therapeutics can only be precisely tested in ex vivo setups due to ethical limitations and pharmacokinetic profiling unlike chemical therapeutics. Our microfluidic platform provided a real-time therapeutic analysis of allogeneic stem cell exosomes in an ex vivo microenvironment. RTCA [79] and microfluidic platforms [59–63] are widely used for drug development in terms of personalized and precise assessment of both cell-based and chemical therapeutics thus our data is reproducible and statistically reliable. Although PT cell lines are questioned in a recent study for resembling native proximal tubules [105], these lines still provide translational tools for diagnostic and therapeutic approaches with help of 3D culture systems [30]. Our platform consists standardized human proximal tubule epithelium (PTE) and BMMSC cell lines that will shed light on future investigations with human primary MSCs and PTE cells. The lack of long-term assessment for AKI-CKD transition is also a limitation mainly due to the challenging long-term maintenance of microfluidic culture setup. Therefore, the results reflect an improvement in the acute phase of kidney injury and the chronic stages of organ failure should be further evaluated. Recently, few preclinical studies reported some mechanisms mediating nephroprotective effects of MSC-Exos such as promotion of tubular proliferation by mRNA of IGF-1 receptor [106] and activation of MAPK/ERK pathway [107, 108], inhibition of apoptosis by upregulating Bcl-2, Bcl-xL and BIRC8 [109] also antioxidation by upregulating Nrf2 [110] and Calbindin1 [111]. Immunomodulation by downregulation of pro-inflammatory cytokines (IL-6, IL-1β, IFN-γ, TNF-α) [18] and promotion of angiogenesis by VEGF-A and bFGF [108] present alternative molecular pathways for therapeutic potential for exosomal products. Those will be the main subject of the future preclinical studies before the clinical phase trials. Thus, present study should continue with molecular scale-up trials on microfluidic 3D platforms comprising larger groups of patients and donor samples [31, 32]. 

In conclusion, our 3D gravity-driven microfluidic acute hypoxic PT injury platform provides a stepping stone to assess pathological mechanisms precisely and evaluate potential theragnostic applications for reaching the goals of 0by25 initiative of ISN. Two-step evaluation of hBMMSC-Exos as a potential therapeutic agent with our novel proliferative potency assay and 3D gravity-driven microfluidic acute hypoxic tubular injury platform provides a reproducible and safety-driven approach against cell-based therapies. The novel system also provides an alternative translational tool to reduce dependency on animal experiments with its controlled microenvironment allowing a realistic modelling for physiological and pathological conditions. The hBMMSC-Exos may provide an allogeneic or autologous intervention tool for AKI-CKD transition by preventing maladaptive repair of the PT epithelium. Future modifications of our platform with patient-derived PTE cells may also serve as a point-of-care evaluation tool for therapeutic efficacy. Assessment of the cell-based therapies on these personalized platforms will provide reassurance about the safety and efficacy of the therapy before clinical use in nephrology.

### Electronic supplementary material

Below is the link to the electronic supplementary material.

Supplementary Material 1

## Data Availability

The statistical numeric data and figures supporting the findings of this study are openly available in Figshare repository at figshare.com [112], 10.6084/m9.figshare.24511363. Additional data are available from the corresponding author upon reasonable request.

## Abbreviations

AKI: Acute kidney injury
PT: Proximal tubule
PTE: Proximal tubule epithelium
CKD: Chronic kidney disease
MSC: Mesenchymal stem cells
hBMMSC: Human bone marrow mesenchymal stem cells
Exos: Exosomes
2D: Two-dimensional
3D: Three-dimensional
RTCA: Real time cell analysis
ZO-1: Zonula occludens 1
WST-1: Water-soluble tetrazolium salt 1
ED50: Median effective dose
ISN: International Nephrology Society
HO-1: Heme oxygenase 1
KIM-1: Kidney injury molecule 1
PDMS: Polydimethylsiloxane
FITC: Fluorescein isothiocyanate
TRITC: Tetramethyl rhodamine
CTFI: Corrected total fluorescence intensity
DMEM: Dulbecco’s modified eagle medium
FBS: Fetal bovine serum
PBS: Phosphate-buffered saline
BCA: Bicinchoninic acid
TEM: Transmission electron microscopy
GFB: Glomerular filtration barrier

## References

[1] Pefanis A, Ierino FL, Murphy JM, Cowan PJ (2019). Regulated necrosis in kidney ischemia-reperfusion injury. Kidney Int.

[2] Liu BC, Tang TT, Lv LL, Lan HY (2018). Renal tubule injury: a driving force toward chronic kidney disease. Kidney Int.

[3] Ullah MM, Basile DP (2019). Role of renal hypoxia in the Progression from Acute kidney Injury to chronic kidney disease. Semin Nephrol.

[4] Han SJ, Lee HT (2019). Mechanisms and therapeutic targets of ischemic acute kidney injury. Kidney Res Clin Pr.

[5] Coca SG, Singanamala S, Parikh CR (2012). Chronic kidney disease after acute kidney injury: a systematic review and meta-analysis. Kidney Int.

[6] Mehta RL, Cerdá J, Burdmann EA, Tonelli M, García-García G, Jha V (2015). International Society of Nephrology’s 0by25 initiative for acute kidney injury (zero preventable deaths by 2025): a human rights case for nephrology. Lancet.

[7] Li W, Duan A, Xing Y, Xu L, Yang J (2021). Transcription-based multidimensional regulation of fatty acid metabolism by HIF1alpha in renal tubules. Front Cell Dev Biol.

[8] Keele GR, Prokop JW, He H, Holl K, Littrell J, Deal AW (2021). Sept8/SEPTIN8 involvement in cellular structure and kidney damage is identified by genetic mapping and a novel human tubule hypoxic model. Sci Rep.

[9] Yu W, Mao QF (2021). Inhibition of TRAF1 protects renal tubular epithelial cells against hypoxia/reoxygenation injury. J Mens Health.

[10] Tang TT, Lv LL, Pan MM, Wen Y, Wang B, Li ZL (2018). Hydroxychloroquine attenuates renal ischemia/reperfusion injury by inhibiting cathepsin mediated NLRP3 inflammasome activation. Cell Death Dis.

[11] Guo SD, Chen WJ, Zheng M, Wang SG (2016). Protective effects of propofol on rat renal tubule epithelial cell line NRK-52E with hypoxia-reoxygenation. Int J Clin Exp Pathol.

[12] Wang Z, Guan W, Han Y, Ren H, Tang X, Zhang H (2015). Stimulation of dopamine D3 receptor attenuates renal ischemia-reperfusion Injury via increased linkage with Galpha12. Transplantation.

[13] Zhao WY, Han S, Zhang L, Zhu YH, Wang LM, Zeng L (2013). Mitochondria-targeted antioxidant peptide SS31 prevents hypoxia/reoxygenation-induced apoptosis by down-regulating p66Shc in renal tubular epithelial cells. Cell Physiol Biochem.

[14] Biju MP, Akai Y, Shrimanker N, Haase VH (2005). Protection of HIF-1-deficient primary renal tubular epithelial cells from hypoxia-induced cell death is glucose dependent. Am J Physiol Ren Physiol.

[15] Cargill KR, Chiba T, Murali A, Mukherjee E, Crinzi E, Sims-Lucas S (2020). Prenatal hypoxia increases susceptibility to kidney injury. PLoS ONE.

[16] Guan P, Sun ZM, Luo LF, Zhao YS, Yang SC, Yu FY et al. Hydrogen Gas alleviates chronic intermittent Hypoxia-Induced Renal Injury through reducing Iron overload. Molecules. 2019;24(6). 10.3390/molecules24061184 PMC6471060 30917568

[17] Wang C, Zhu G, He W, Yin H, Lin F, Gou X (2019). BMSCs protect against renal ischemia-reperfusion injury by secreting exosomes loaded with miR-199a-5p that target BIP to inhibit endoplasmic reticulum stress at the very early reperfusion stages. FASEB J.

[18] Alzahrani FA (2019). Melatonin improves therapeutic potential of mesenchymal stem cells-derived exosomes against renal ischemia-reperfusion injury in rats. Am J Transl Res.

[19] Soo JY, Jansen J, Masereeuw R, Little MH (2018). Advances in predictive in vitro models of drug-induced nephrotoxicity. Nat Rev Nephrol.

[20] Sadeghian RB, Ueno R, Araoka T, Yamashita J, Enoki T, Takasato M et al. In. Effect of Perfusion Culture on localization, intensity, and functionality of Transporter proteins in a Bilayer Proximal Tubule-on-a chip. 2021.

[21] Nieskens TT, Wilmer MJ (2016). Kidney-on-a-chip technology for renal proximal tubule tissue reconstruction. Eur J Pharmacol.

[22] Wilmer MJ, Ng CP, Lanz HL, Vulto P, Suter-Dick L, Masereeuw R (2016). Kidney-on-a-Chip Technology for Drug-Induced Nephrotoxicity Screening. Trends Biotechnol.

[23] Zhang XF, Wang N, Huang YH, Li Y, Li G, Lin YX et al. Extracellular vesicles from three dimensional culture of human placental mesenchymal stem cells ameliorated renal ischemia/reperfusion injury. Int J Artif Organs. 2021;12. 10.1177/0391398820986809 33467948

[24] Zhao M, Liu SY, Wang CS, Wang YZ, Wan MH, Liu F (2021). Mesenchymal stem cell-derived extracellular vesicles attenuate mitochondrial damage and inflammation by stabilizing mitochondrial DNA. ACS Nano.

[25] Inotani S, Taniguchi Y, Nakamura K, Nishikawa H, Matsumoto T, Horino T (2022). Knockout of Zeb2 ameliorates progression of renal tubulointerstitial fibrosis in a mouse model of renal ischemia-reperfusion injury. Nephrol Dial Transpl off Publ Eur Dial Transpl Assoc - Eur Ren Assoc.

[26] Coux G, Elías MM, Trumper L (2009). Ischaemia/reperfusion in rat renal cortex: vesicle leakiness and Na+, K+-ATPase activity in membrane preparations. Nephrol Dial Transplant off Publ Eur Dial Transpl Assoc. Eur Ren Assoc.

[27] Shang ZZ, Jiang YB, Guan X, Wang AA, Ma B (2021). Therapeutic effects of Stem cells from different source on Renal Ischemia- Reperfusion Injury: a systematic review and network Meta-analysis of Animal studies. Front Pharmacol.

[28] McCafferty K, Forbes S, Thiemermann C, Yaqoob MM (2014). The challenge of translating ischemic conditioning from animal models to humans: the role of comorbidities. Dis Model Mech.

[29] Singh AP, Muthuraman A, Jaggi AS, Singh N, Grover K, Dhawan R (2012). Animal models of acute renal failure. Pharmacol Rep.

[30] Ingber DE (2020). Is it time for reviewer 3 to request human organ chip experiments instead of animal validation studies?. Adv Sci Weinh.

[31] Willis GR, Kourembanas S, Mitsialis SA (2017). Toward exosome-based therapeutics: isolation, heterogeneity, and fit-for-purpose potency. Front Cardiovasc Med.

[32] Fazekas B, Griffin MD (2020). Mesenchymal stromal cell ? Based therapies for acute kidney injury: progress in the last decade. Kidney Int.

[33] Tseng WC, Lee PY, Tsai MT, Chang FP, Chen NJ, Chien CT (2021). Hypoxic mesenchymal stem cells ameliorate acute kidney ischemia-reperfusion injury via enhancing renal tubular autophagy. Stem Cell Res Ther.

[34] Zhou S, Qiao YM, Liu YG, Liu D, Hu JM, Liao J (2020). Bone marrow derived mesenchymal stem cells pretreated with erythropoietin accelerate the repair of acute kidney injury. Cell Biosci.

[35] Shen B, Liu J, Zhang F, Wang Y, Qin Y, Zhou ZH (2016). CCR2 positive exosome released by mesenchymal stem cells suppresses macrophage functions and alleviates Ischemia/Reperfusion-Induced Renal Injury. Stem Cells Int.

[36] Massa M, Croce S, Campanelli R, Abba C, Lenta E, Valsecchi C (2020). Clinical applications of mesenchymal Stem/Stromal cell derived Extracellular vesicles: therapeutic potential of an Acellular product. Diagnostics.

[37] Nargesi AA, Lerman LO, Eirin A (2017). Mesenchymal stem cell-derived extracellular vesicles for kidney repair: current status and looming challenges. Stem Cell Res Ther.

[38] Moghadasi S, Elveny M, Rahman HS, Suksatan W, Jalil AT, Abdelbasset WK (2021). A paradigm shift in cell-free approach: the emerging role of MSCs-derived exosomes in regenerative medicine. J Transl Med.

[39] Yun CW, Lee SH (2019). Potential and therapeutic efficacy of cell-based Therapy using mesenchymal stem cells for Acute/chronic kidney disease. Int J Mol Sci.

[40] Lee PW, Wu BS, Yang CY, Lee OK (2021). Molecular mechanisms of mesenchymal stem cell-based therapy in Acute kidney Injury. Int J Mol Sci.

[41] Tögel FE, Westenfelder C (2012). Kidney protection and regeneration following acute injury: progress through stem cell therapy. Am J Kidney Dis.

[42] Cao JY, Wang B, Tang TT, Wen Y, Li ZL, Feng ST (2021). Exosomal miR-125b-5p deriving from mesenchymal stem cells promotes tubular repair by suppression of p53 in ischemic acute kidney injury. Theranostics.

[43] Zhang ZY, Hou YP, Zou XY, Xing XY, Ju GQ, Zhong L (2020). Oct-4 enhanced the Therapeutic effects of mesenchymal stem cell-derived extracellular vesicles in Acute kidney Injury. Kidney Blood Press Res.

[44] Zhu G, Pei L, Lin F, Yin H, Li X, He W (2019). Exosomes from human-bone-marrow-derived mesenchymal stem cells protect against renal ischemia/reperfusion injury via transferring miR-199a-3p. J Cell Physiol.

[45] Zhou YJ, Liu SY, Zhao M, Wang CS, Li L, Yuan YJ (2019). Injectable extracellular vesicle-released self-assembling peptide nanofiber hydrogel as an enhanced cell-free therapy for tissue regeneration. J Controlled Release.

[46] Zhang C, Shang Y, Chen X, Midgley AC, Wang Z, Zhu D (2020). Supramolecular nanofibers containing arginine-Glycine-aspartate (RGD) peptides boost therapeutic efficacy of Extracellular vesicles in kidney repair. ACS Nano.

[47] Zhu FM, Shin O, Pei GC, Hu ZZ, Yang J, Zhu H (2017). Adipose-derived mesenchymal stem cells employed exosomes to attenuate AKI-CKD transition through tubular epithelial cell dependent Sox9 activation. Oncotarget.

[48] Collino F, Pomatto M, Bruno S, Lindoso RS, Tapparo M, Sicheng W (2017). Exosome and Microvesicle-enriched fractions isolated from mesenchymal stem cells by gradient separation showed different molecular signatures and functions on renal tubular epithelial cells. Stem Cell Rev Rep.

[49] Dou M, Guo Y, Zheng B, Li Y, Zheng J, Wang B (2023). Exsomal microRNA-223 attenuates pyroptosis in Renal Ischemia/Reperfusion Injury by Targeting NLR Family Pyrin Domain containing 3. JOURNAL OF BIOLOGICAL REGULATORS AND HOMEOSTATIC AGENTS. VIA S STEFANO 39 BIS, 64029 SILVA MARINA (TE).

[50] Altun B, Yilmaz R, Aki T, Akoglu H, Zeybek D, Piskinpasa S (2012). Use of mesenchymal stem cells and Darbepoetin improve Ischemia-Induced Acute kidney Injury outcomes. Am J Nephrol.

[51] Lim SW, Kim KW, Kim BM, Shin YJ, Luo K, Quan Y et al. Alleviation of renal ischemia/reperfusion injury by exosomes from induced pluripotent stem cell-derived mesenchymal stem cells. Korean J Intern Med. 2021. 10.3904/kjim.2020.438 PMC8925954 34521186

[52] Zhang G, Wang D, Miao S, Zou X, Liu G, Zhu Y (2016). Extracellular vesicles derived from mesenchymal stromal cells may possess increased therapeutic potential for acute kidney injury compared with conditioned medium in rodent models: a meta-analysis. Exp Ther Med.

[53] Zhang YQ, Wang CJ, Bai ZX, Li P (2021). Umbilical cord mesenchymal stem cell exosomes alleviate the progression of kidney failure by modulating inflammatory responses and oxidative stress in an ischemia-reperfusion mice Model. J Biomed Nanotechnol.

[54] Sun Z, Wu J, Bi Q, Wang W. Exosomal lncRNA TUG1 derived from human urine-derived stem cells attenuates renal ischemia/reperfusion injury by interacting with SRSF1 to regulate ASCL4-mediated ferroptosis. STEM CELL Res Ther. 2022;13(1). 10.1186/s13287-022-02986-x PMC9284726 35841017

[55] García-Ortuño LE, Bobadilla NA (2018). Integrative view of the mechanisms that induce acute kidney Injury and its transition to chronic kidney disease. Rev Invest Clin.

[56] Maekawa H, Inagi R (2019). Pathophysiological Role of Organelle Stress/Crosstalk in AKI-to-CKD transition. Semin Nephrol.

[57] Cao HM, Cheng YQ, Gao HQ, Zhuang J, Zhang WG, Bian Q (2020). In vivo Tracking of mesenchymal stem cell-derived extracellular vesicles improving mitochondria! Function in Renal Ischemia- Reperfusion Injury. ACS Nano.

[58] Zhang KY, Chen S, Sun HM, Wang LN, Li HF, Zhao JL (2020). In vivotwo-photon microscopy reveals the contribution of Sox9(+)cell to kidney regeneration in a mouse model with extracellular vesicle treatment. J Biol Chem.

[59] Rothbauer M, Bachmann BEM, Eilenberger C, Kratz SRA, Spitz S, Holl G et al. A decade of organs-on-a-Chip emulating human physiology at the Microscale: a critical Status Report on Progress in Toxicology and Pharmacology. Micromachines Basel. 2021;12(5). 10.3390/mi12050470 PMC8143089 33919242

[60] Ashammakhi N, Wesseling-Perry K, Hasan A, Elkhammas E, Zhang YS (2018). Kidney-on-a-chip: untapped opportunities. Kidney Int.

[61] Peired AJ, Mazzinghi B, De Chiara L, Guzzi F, Lasagni L, Romagnani P (2020). Bioengineering strategies for nephrologists: kidney was not built in a day. Expert Opin Biol Ther.

[62] Cong Y, Han X, Wang Y, Chen Z, Lu Y, Liu T et al. Drug toxicity evaluation based on organ-on-a-chip Technology: a review. Micromachines Basel. 2020;11(4). 10.3390/mi11040381 PMC7230535 32260191

[63] Ramadan Q, Zourob M (2020). Organ-on-a-chip engineering: toward bridging the gap between lab and industry. Biomicrofluidics.

[64] Xiong S, Tan S, Huang P, Li Y, Chung JE, Kurisawa M (2023). Toxicity and efficacy of green tea catechin derivative-based micellar nanocomplexes for anticancer protein delivery. Biomater Sci.

[65] Adler M, Ramm S, Hafner M, Muhlich JL, Gottwald EM, Weber E (2016). A quantitative Approach to screen for Nephrotoxic compounds in Vitro. J Am Soc Nephrol.

[66] Maass C, Sorensen NB, Himmelfarb J, Kelly EJ, Stokes CL, Cirit M (2019). Translational Assessment of Drug-Induced Proximal Tubule Injury using a kidney Microphysiological System. CPT Pharmacomet Syst Pharmacol.

[67] Weber EJ, Lidberg KA, Wang L, Bammler TK, MacDonald JW, Li MJ et al. Human kidney on a chip assessment of polymyxin antibiotic nephrotoxicity. JCI Insight. 2018;3(24). 10.1172/jci.insight.123673 PMC6338315 30568031

[68] Jang KJ, Mehr AP, Hamilton GA, McPartlin LA, Chung S, Suh KY (2013). Human kidney proximal tubule-on-a-chip for drug transport and nephrotoxicity assessment. Integr Biol.

[69] Vormann MK, Gijzen L, Hutter S, Boot L, Nicolas A, van den Heuvel A (2018). Nephrotoxicity and Kidney Transport Assessment on 3D perfused proximal tubules. Aaps J.

[70] Vriend J, Nieskens TTG, Vormann MK, van den Berge BT, van den Heuvel A, Russel FGM (2018). Screening of drug-transporter interactions in a 3D microfluidic renal proximal tubule on a chip. Aaps J.

[71] Vriend J, Peters JGP, Nieskens TTG, Skovronova R, Blaimschein N, Schmidts M (2020). Flow stimulates drug transport in a human kidney proximal tubule-on-a-chip independent of primary cilia. Biochim Biophys Acta Gen Subj.

[72] Suter-Dick L, Mauch L, Ramp D, Caj M, Vormann MK, Hutter S (2018). Combining Extracellular miRNA determination with microfluidic 3D cell cultures for the Assessment of Nephrotoxicity: a Proof of Concept Study. AAPS J.

[73] Vormann MK, Vriend J, Lanz HL, Gijzen L, van den Heuvel A, Hutter S (2021). Implementation of a human renal proximal tubule on a chip for Nephrotoxicity and Drug Interaction studies. J Pharm Sci.

[74] Vriend J, Vormann MK, Lanz HL, Joore J, Trietsch SJ, Russel FGM (2021). Nephroscreen: a robust and versatile renal tubule-on-a-chip platform for nephrotoxicity assessment. Curr Opin Toxicol.

[75] Hargrove-Grimes P, Low LA, Tagle DA (2021). Microphysiological systems: what it takes for community adoption. Exp Biol Med Maywood.

[76] Wang Y, Gao Y, Pan Y, Zhou D, Liu Y, Yin Y (2023). Emerging trends in organ-on-a-chip systems for drug screening. ACTA Pharm Sin B.

[77] Sunildutt N, Parihar P, Salih ARC, Lee SH, Choi KH (2023). Revolutionizing drug development: harnessing the potential of organ-on-chip technology for disease modeling and drug discovery. Front Pharmacol.

[78] Collino F, Bruno S, Incarnato D, Dettori D, Neri F, Provero P (2015). AKI Recovery Induced by Mesenchymal Stromal Cell-Derived Extracellular vesicles carrying MicroRNAs. J Am Soc Nephrol.

[79] Yersal N, Kose S, Horzum U, Ozkavukcu S, Orwig KE, Korkusuz P (2020). Leptin promotes proliferation of neonatal mouse stem/progenitor spermatogonia. J Assist Reprod Genet.

[80] Théry C, Witwer KW, Aikawa E, Alcaraz MJ, Anderson JD, Andriantsitohaina R (2018). Minimal information for studies of extracellular vesicles 2018 (MISEV2018): a position statement of the International Society for Extracellular Vesicles and update of the MISEV2014 guidelines. J Extracell Vesicles.

[81] Eroglu FK, Yazar V, Guler U, Yıldırım M, Yildirim T, Gungor T (2021). Circulating extracellular vesicles of patients with steroid-sensitive nephrotic syndrome have higher RAC1 and induce recapitulation of nephrotic syndrome phenotype in podocytes. Am J Physiol-Ren Physiol.

[82] Li ZY, Zhu JN, Wan ZH, Li GH, Chen L, Guo YL. Theaflavin ameliorates renal ischemia/reperfusion injury by activating the Nrf2 signalling pathway in vivo and in vitro. Biomed Pharmacother. 2021;134. 10.1016/j.biopha.2020.111097 33341051

[83] Lawrence MG, Altenburg MK, Sanford R, Willett JD, Bleasdale B, Ballou B (2017). Permeation of macromolecules into the renal glomerular basement membrane and capture by the tubules. Proc Natl Acad Sci U A.

[84] Englezakis A, Gozalpour E, Kamran M, Fenner K, Mele E, Coopman K (2021). Development of a hollow fibre-based renal module for active transport studies. J Artif Organs.

[85] Nieskens TTG, Persson M, Kelly EJ, Sjogren AK (2020). A Multicompartment human kidney proximal tubule-on-a-Chip replicates cell polarization-dependent cisplatin toxicity. Drug Metab Dispos.

[86] Otani T, Furuse M (2020). Tight Junction structure and function revisited. Trends Cell Biol.

[87] Cantaluppi V, Medica D, Quercia AD, Dellepiane S, Figliolini F, Virzi GM (2018). Perfluorocarbon solutions limit tubular epithelial cell injury and promote CD133(+) kidney progenitor differentiation: potential use in renal assist devices for sepsis-associated acute kidney injury and multiple organ failure. Nephrol Dial Transpl.

[88] Ciftci E, Bozbeyoglu N, Gursel I, Korkusuz F, Misirlioglu FB, Korkusuz P (2023). Comparative analysis of magnetically activated cell sorting and ultracentrifugation methods for exosome isolation. PLoS ONE.

[89] Tracy SA, Ahmed A, Tigges JC, Ericsson M, Pal AK, Zurakowski D (2019). A comparison of clinically relevant sources of mesenchymal stem cell-derived exosomes: bone marrow and amniotic fluid. J Pediatr Surg.

[90] Chen J, Chen J, Cheng Y, Fu Y, Zhao H, Tang M (2020). Mesenchymal stem cell-derived exosomes protect beta cells against hypoxia-induced apoptosis via miR-21 by alleviating ER stress and inhibiting p38 MAPK phosphorylation. Stem Cell Res Ther.

[91] Sung SE, Seo MS, Kang KK, Choi JH, Lee SJ, Lim JH et al. Isolation and characterization of Extracellular Vesicle from Mesenchymal Stem cells of the Epidural Fat of the spine. Asian Spine J. 2021. 10.31616/asj.2021.0129 PMC9066249 34461688

[92] Yuan X, Li D, Chen X, Han C, Xu L, Huang T (2017). Extracellular vesicles from human-induced pluripotent stem cell-derived mesenchymal stromal cells (hiPSC-MSCs) protect against renal ischemia/reperfusion injury via delivering specificity protein (SP1) and transcriptional activating of sphingosine kinase 1 and inhibiting necroptosis. Cell Death Dis.

[93] Zahran R, Ghozy A, Elkholy SS, El-Taweel F, El-Magd MA (2020). Combination therapy with melatonin, stem cells and extracellular vesicles is effective in limiting renal ischemia-reperfusion injury in a rat model. Int J Urol.

[94] Shekari F, Nazari A, Assar Kashani S, Hajizadeh-Saffar E, Lim R, Baharvand H (2021). Pre-clinical investigation of mesenchymal stromal cell-derived extracellular vesicles: a systematic review. Cytotherapy.

[95] Dal Collo G, Adamo A, Gatti A, Tamellini E, Bazzoni R, Takam Kamga P (2020). Functional dosing of mesenchymal stromal cell-derived extracellular vesicles for the prevention of acute graft-versus-host-disease. Stem Cells.

[96] Pachler K, Ketterl N, Desgeorges A, Dunai ZA, Laner-Plamberger S, Streif D et al. An in Vitro Potency Assay for Monitoring the Immunomodulatory potential of stromal cell-derived extracellular vesicles. Int J Mol Sci. 2017;18(7). 10.3390/ijms18071413 PMC5535905 28671586

[97] Teryek M, Doshi A, Sherman LS, Rameshwar P, Jung S, Parekkadan B. Clinical Manufacturing of Human Mesenchymal Stromal Cells using a potency-driven paradigm. Curr Stem Cell Rep. 2022.

[98] Simonsen JB. Pitfalls associated with lipophilic fluorophore staining of extracellular vesicles for uptake studies. J Extracell Vesicles. 2019;8(1). 10.1080/20013078.2019.1582237 PMC6383605 30815239

[99] Morishita M, Takahashi Y, Nishikawa M, Sano K, Kato K, Yamashita T (2015). Quantitative Analysis of Tissue Distribution of the B16BL6-Derived exosomes using a Streptavidin-Lactadherin Fusion protein and iodine-125-Labeled biotin derivative after intravenous injection in mice. J Pharm Sci.

[100] Jung KO, Youn H, Lee CH, Kang KW, Chung JK. Visualization of exosome-mediated miR-210 transfer from hypoxic tumor cells. Oncotarget. 2016;8(6). 10.18632/oncotarget.14247 PMC5354779 28038441

[101] Pan Q, Kuang X, Cai S, Wang X, Du D, Wang J (2020). Mir-132-3p priming enhances the effects of mesenchymal stromal cell-derived exosomes on ameliorating brain ischemic injury. Stem Cell Res Ther.

[102] He S, Liu G, Zhu X (2021). Human breast milk-derived exosomes may help maintain intestinal epithelial barrier integrity. Pediatr Res.

[103] Lin N, Zhou X, Geng X, Drewell C, Hubner J, Li Z (2020). Repeated dose multi-drug testing using a microfluidic chip-based coculture of human liver and kidney proximal tubules equivalents. Sci Rep.

[104] Sakolish CM, Philip B, Mahler GJ (2019). A human proximal tubule-on-a-chip to study renal disease and toxicity. Biomicrofluidics.

[105] Khundmiri SJ, Chen L, Lederer ED, Yang CR, Knepper MA (2021). Transcriptomes of Major Proximal Tubule Cell Culture models. J Am Soc Nephrol JASN.

[106] Tomasoni S, Longaretti L, Rota C, Morigi M, Conti S, Gotti E (2013). Transfer of growth factor receptor mRNA via exosomes unravels the regenerative effect of mesenchymal stem cells. Stem Cells Dev.

[107] Ullah M, Liu DD, Rai S, Razavi M, Choi J, Wang J et al. A Novel Approach to deliver therapeutic extracellular vesicles directly into the mouse kidney via its arterial blood supply. Cells. 2020;9(4). 10.3390/cells9040937 PMC7226986 32290286

[108] Ullah M, Liu DD, Rai S, Razavi M, Concepcion W, Thakor AS (2020). Pulsed focused ultrasound enhances the therapeutic effect of mesenchymal stromal cell-derived extracellular vesicles in acute kidney injury. Stem Cell Res Ther.

[109] Zhou Y, Xu HT, Xu WR, Wang BY, Wu HY, Tao Y (2013). Exosomes released by human umbilical cord mesenchymal stem cells protect against cisplatin-induced renal oxidative stress and apoptosis in vivo and in vitro. Stem Cell Res Ther.

[110] Zhang G, Zou X, Huang Y, Wang F, Miao S, Liu G (2016). Mesenchymal stromal cell-derived extracellular vesicles protect against acute kidney Injury through Anti-oxidation by enhancing Nrf2/ARE activation in rats. Kidney Blood Press Res.

[111] Gregorini M, Corradetti V, Pattonieri EF, Rocca C, Milanesi S, Peloso A (2017). Perfusion of isolated rat kidney with mesenchymal stromal Cells/Extracellular vesicles prevents ischaemic injury. J Cell Mol Med.

[112] Sefa Burak Çam E, Ciftci. Nazlıhan Gürbüz, Bülent Altun, Petek Korkusuz. Exosomes on Hypoxic AKI-on-a-Chip [Internet]. 2023. Available from: https://figshare.com/articles/dataset/Exosomes_on_Hypoxic_AKI-on-a-Chip/24511363. 10.1186/s13287-024-03674-8 PMC11005291 38600585

