# Supplementary material for: Allogeneic bone marrow mesenchymal stem cell-derived exosomes alleviate human hypoxic AKI-on-a-Chip within a tight treatment window
Source: Stem Cell Res Ther. 2024 Apr 10;15:105. doi: 10.1186/s13287-024-03674-8 (PMC11005291; doi:10.1186/s13287-024-03674-8)
Supplement: Supplementary file 1 — Supplementary Material 1 [file 13287_2024_3674_MOESM1_ESM.docx]

Rocky S Tuan, PhD, Editor-in-Chief,

Stem Cell Research & Therapy

January 18, 2024

Dear Rocky,

I wish you and your family a happy and prosperous New Year. May this new year be filled with professional growth, personal fulfillment, and the joy that comes from making a meaningful impact together.

We would be honored to hear that our manuscript entitled “**Allogeneic Bone Marrow Mesenchymal Stem Cell-Derived Exosomes Alleviate Human Hypoxic AKI-on-a-Chip in a Tight Treatment Window**” has been accepted for reconsideration with major revisions for publication in Stem Cell Research & Therapy for the specific collection on Organoids and Tissue/Organ Chips. We have carefully taken the comments into consideration, answered them one by one in our revision.

We revisited the Guidelines for exosome characterization criteria, revised the Method, Results, Figures and improved the Discussion section according to the reviewers’ valuable comments in our final manuscript. We have submitted the final and highlighted version of the manuscript and related files.

As the corresponding author of the current publication, I would like to remind you again that we do not have any ongoing support available for covering the publication fee and open access funds. The documentation of my funding status and the letter of the funding coordinator have been provided previously. Thus, we kindly request you to waive the publication charges and give us the opportunity to publish our paper in Stem Cell Research & Therapy.

We are looking forward reading from you soon.

Petek Korkusuz, M.D., Ph.D.,

Professor of Histology and Embryology

Hacettepe University Faculty of Medicine

Department of Histology and Embryology

Sihhiye, 06230 Ankara, Turkey

Phone: +90-312-3052165

Mobile: +90-532-2968138

e-mail: [petek@hacettepe.edu.tr](mailto:petek@hacettepe.edu.tr)
